# Parametric Study of Effects of Atmospheric Pressure Plasma Treatment on the Wettability of Cotton Fabric

**DOI:** 10.3390/polym10030233

**Published:** 2018-02-26

**Authors:** Chi-Wai Kan, Wai-Shan Man

**Affiliations:** Institute of Textiles and Clothing, The Hong Kong Polytechnic University, Hung Hom, Kowloon, Hong Kong, China; wsman@yahoo.com

**Keywords:** atmospheric pressure plasma, cotton, wettability, wicking, parameter

## Abstract

In textiles processing, wettability of fabric plays a very important role in enhancing processes such as dyeing and printing. Although well-prepared cotton fabric has very good wettability, further enhancement of its wettability can effectively improve the subsequent dyeing and printing processes. Plasma treatment, especially atmospheric pressure plasma treatment (APPT), a continuous process, is now drawing attention of the industry. In this study, we investigated the effect of APPT under four operational parameters: (1) discharge power; (2) flow rate of oxygen; (3) jet travelling speed; and (4) jet-to-substrate distance on wettability (in terms of wickability and wetting area) of cotton fabric. Experimental results revealed that the four parameters interact with each other in affecting the wettability of the cotton fabric. The results are discussed comprehensively.

## 1. Introduction

Plasma modification of textile-based material is based on the interaction between the substrate and active species in the plasma. Generally speaking, active plasma species react with the material surface without modifying bulk properties of the materials [1]. The plasma modification mechanism is significantly affected by treatment conditions such as gas flow rate, nature of gas used and discharge power [2]. The effect of plasma treatment on the substrate is highly dependent upon the nature of gas feeding into the plasma process gas [1,3]. Polymerizing and non-polymerizing gases can be used for plasma treatment depending on the final surface properties required. Polymerizing gases used in plasma treatment contain carbon and hydrogen atoms such as methane, ethylene and ethanol [4]. They may be polymerized under the effect of plasma treatment. On the other hand, non-polymerizing gases like noble gas, nitrogen, oxygen, hydrogen and ammonia can modify polymer surface through different reactions such as oxidation, ablation, etching, crosslinking and grafting. It should be mentioned that the nature of gas feeding into the plasma leads to specific chemical reactions on the substrate surface [1].

For textile applications, atmospheric pressure plasma treatment, which can be used as a continuous treatment process, has recently drawn much attention from researchers and the industry. Table 1 shows a comparison between plasma treated and conventional chemically processed textiles.

Plasma-modified textile materials show enhanced wettability and hydrophilicity after insertion of functional groups such as carbonyl (–C=O), carboxylic acid (–COOH), hydroxyl (–OH) and/or amine (–NH_2_) groups [6,7,8,9,10] or increased hydrophobicity on removal of hydrophilic functional groups or changing hydrophilic groups into non-hydrophilic groups [11,12]. In addition, removal of hydrophobic layer improves the dyeability [13,14,15,16]. Plasma treatment can reduce shrinkage [17] and improve adhesion of different chemical finishing materials [18,19,20,21]. Therefore, in this study, we investigate systematically the effect of the following atmospheric pressure plasma operational parameters: (i) discharge power; (ii) oxygen flow rate; (iii) jet travelling speed; and (iv) jet-to-substrate distance, on wettability of cotton fabric. This study can help to explore the possible application with systematic study of operational parameters of atmospheric pressure plasma treatment in textile usage such as pigment dyeing and coating.

## 2. Experimental

### 2.1. Cotton Fabric

In this study, 100% ready-for-dyeing plain weave cotton fabric was used [22]. The fabric was washed for 5 min. with diluted acetone (Reagent Grade of 99% purity) and subsequently completely dried in oven at 50 °C. Samples were conditioned at 20 ± 2 °C temperature and relative humidity of 65 ± 2% for at least 24 h before use. 

### 2.2. Atmospheric Pressure Plasma Treatment

**Figure 1:**
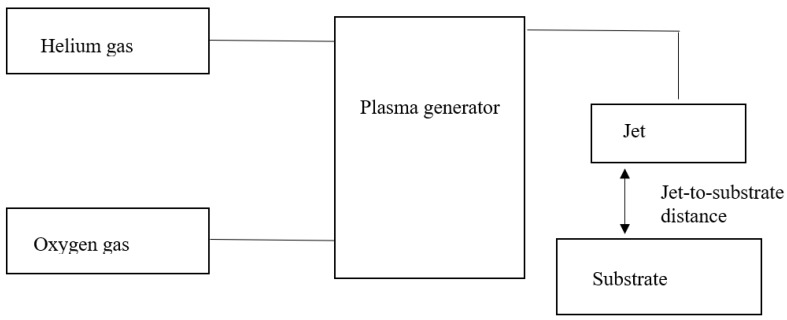
Schematic diagram of set-up of atmospheric pressure plasma treatment system.

Atmospheric pressure plasma treatment of cotton fabric was conducted by a pressure plasma jet (APPJ, Atomflo 400, AH-550L, Surfx Technologies LLC, Redondo, CA, USA) mentioned in previous work [22]. The set-up of plasma treatment is schematically shown in Figure 1. Oxygen gas (99.7% purity) was used as reactive gas while helium (99.995% purity) was used as the carrier gas for the atmospheric pressure plasma treatment. The helium flow rate was fixed at 30 L/min. Four operational parameters were used in this study: (1) discharge power (130 W, 140 W, 150 W, 160 W and 170 W); (2) oxygen flow rate (0.2 L/min, 0.3 L/min, 0.4 L/min, 0.5 L/min and 0.6 L/min); (3) jet travelling speed (1 mm/s, 3 mm/s, 5 mm/s, 7 mm/s and 9 mm/s); and (4) jet-to-substrate distance (3 mm, 4 mm, 5 mm, 7 mm and 9 mm). Effects of different combinations of operational parameters of atmospheric pressure plasma treatment on wettability of cotton fabric were studied; the related combinations are described in the Results and Discussion Section. After atmospheric pressure plasma treatment, the treated fabric was conditioned at 20 ± 2 °C temperature and relative humidity of 65 ± 2% for at least 24 h before measuring the wettability. 

### 2.3. Wicking Rate Measurement

Fabric specimens of size 1.5 cm (width) × 10 cm (length) were cut in warp and weft directions (6 specimens in each direction). Scales of 8 cm were marked in the fabric specimen by water-soluble ink. The fabric specimen was held vertically and the lower edge (weight of 1 g was attached to maintain tension and avoid formation of crease in the fabric specimens during the test) was just immersed in a large volume of distilled water. The time when water reached each graduated scale by capillary force vertically was recorded. Wicking test was conducted for the six fabric specimens in each direction of fabric specimens under standard conditions (temperature: 20 ± 2 °C; relative humidity: 65 ± 2%). The data obtained from wicking test were converted into wicking coefficient by Equation (1). After rearrangement of Equation (1), Equations (2) and (3) show the slope of height (*h*) versus time (*t*^½^) graph and the wicking coefficient (*W_c_*). *W_c_* is used to describe the wicking performance, the higher the *W_c_*, the better is the water absorption ability.

(1) $$h=\sqrt{\frac{r_{c}\gamma \cos\theta t}{2\eta }}=W_{c}\cdot t^{\frac{1}{2}}$$

(2) $$h^{2}=\frac{r_{c}\gamma }{2\eta }\cdot t$$

(3) $$W_{c}=\sqrt{\frac{r_{c}\gamma \cos\theta }{2\eta }}$$

where *h* is height reached by liquid at time *t*; *r_c_* is the effective hydraulic radius of the capillaries; γ is the surface tension of the liquid–vapor interface; θ is the apparent contact angle of the fabric (in vertical wicking test, θ = 180°, cos θ = 1); η is the viscosity of the liquid; *W_c_* is the wicking coefficient; and *t* is the time [23]. 

### 2.4. Drop Test 

After 24 h conditioning, 20 µm of Methylene Blue dye solution was dropped on fabric surface perpendicularly by an autopipette. The area of dispersion of the Methylene Blue dye solution absorbed in fabric was measured after no further spreading was observed. Six measurements were obtained for analyzing the wetting area (mm^2^).

### 2.5. Data Analysis

The measured results were averaged with 95% confidence level to have statistically related data for analysis. 

## 3. Results and Discussion

As a reference point, warp wicking coefficient, weft wicking coefficient and total wicking coefficient of untreated cotton fabric are 24.7, 20.9 and 45.6 respectively, and the wetting area for untreated cotton fabric is 229.8 mm^2^. 

### 3.1. Discharge Power

To investigate the effect of discharge power of atmospheric pressure plasma treatment on wettability of cotton fabric, different discharge powers (130 W, 140 W, 150 W, 160 W and 170 W) and oxygen flow rates (0.2 L/min, 0.3 L/min and 0.4 L/min) were used while other parameters such as jet travelling speed and jet-to-substrate distance were kept at 5 mm/s and 3 mm respectively. 

**Figure 2:**
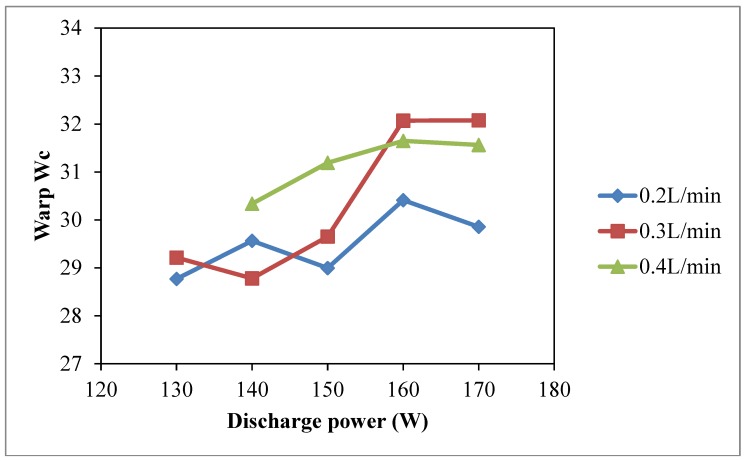
Effect of discharge power on warp *W_c_* (Discharge power: 130 W, 140 W, 150 W, 160 W and 170 W; Oxygen flow rates: 0.2 L/min, 0.3 L/min and 0.4 L/min; Jet travelling speed: 5 mm/s and Jet-to-substrate distance: 3 mm).

**Figure 3:**
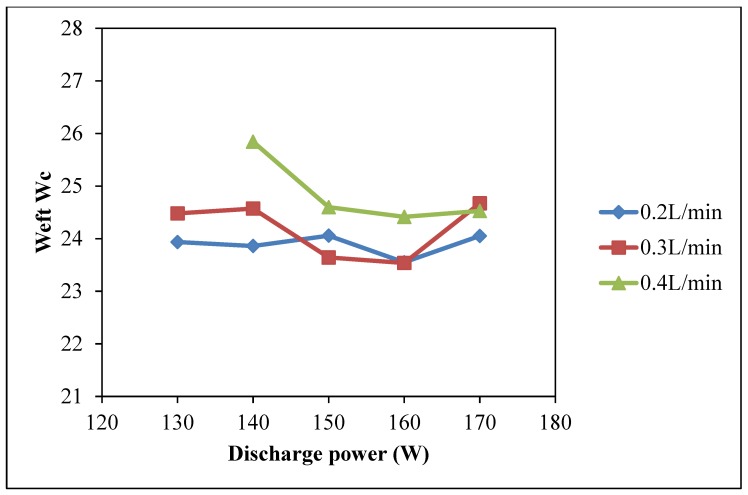
Effect of discharge power on weft *W_c_* (Discharge power: 130 W, 140 W, 150 W, 160 W and 170 W; Oxygen flow rate: 0.2 L/min, 0.3 L/min and 0.4 L/min; Jet travelling speed: 5 mm/s and Jet-to-substrate distance: 3 mm).

**Figure 4:**
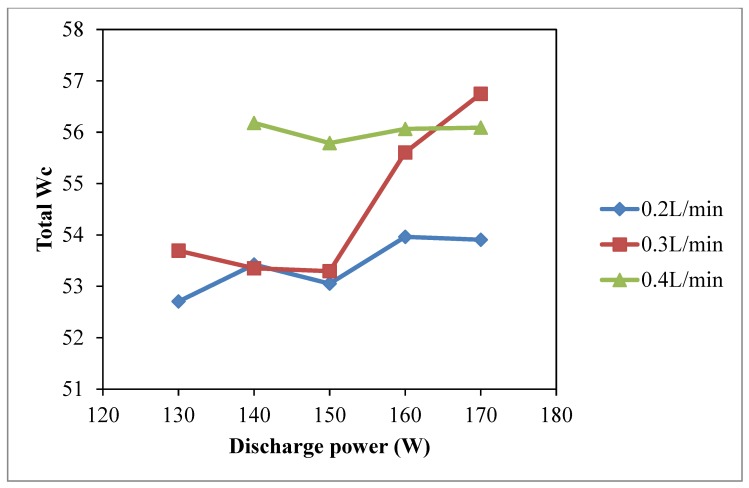
Effect of discharge power on total *W_c_* (Discharge power: 130 W, 140 W, 150 W, 160 W and 170 W; Oxygen flow rate: 0.2 L/min, 0.3 L/min and 0.4 L/min; Jet travelling speed: 5 mm/s and Jet-to-substrate distance: 3 mm).

The warp, weft and total wicking coefficient (*W_c_*), as shown in Figure 2, Figure 3 and Figure 4, respectively, vary with discharge power. The discharge power of 130 W with oxygen flow rate of 0.4 L/min is absent because the machine setting did not allow this combination of parameters since this could produce unstable plasma effect. Compared with the warp *W_c_*, weft *W_c_* and total *W_c_* of untreated cotton fabric, wicking performance of the plasma-treated cotton samples is greatly improved and is better than untreated cotton fabric [23,24]. 

As shown in Figure 2, warp wicking behavior of cotton fabric is directly correlated to the discharge power used in the plasma treatment. A higher value of warp *W_c_* is obtained with a higher discharge power and it is more significant when oxygen flow rate is 0.3 L/min. However, in the weft direction, discharge power induces less wicking effect (Figure 3). However, with oxygen flow rate of 0.4 L/min, better weft *W_c_* values can be obtained compared with oxygen flow rates of 0.2 L/min and 0.3 L/min.

The penetration power of active plasma species depends on discharge energy of plasma treatment and the duration for which they remain active, which implies a more pronounced effect under high discharge power. Since the atmospheric pressure plasma treatment in this study is a one side treatment, if the active plasma species have a short lifetime, they cannot penetrate deep enough to interact with weft yarns of a woven fabric because the weft yarns are covered by the warp yarns. The weft yarns are thus hidden by the warp yarns and plasma effect is less for the weft yarns [9,23]. The increase of weft *W_c_* in low discharge power is due to oxidation of fiber surface when lifespan of free oxygen radicals is longer [9,23]. As a result, the effects of discharge power on wicking in warp (Figure 2) and weft (Figure 3) directions are different. Thus, the total *W_c_* is plotted (Figure 4) which shows that total *W_c_* is increasing as the discharge power is generally increased with different oxygen flow rates. The maximum total *W_c_* is at discharge power of 170 W with oxygen flow rate of 0.3 L/min. A high discharge power generally induces a formation of plasma active species at a higher rate which increases the etching and oxidation effect on the substrate surface [23]. Moreover, active plasma species possess a higher energy that can interact with the substrate because high discharge power can supply more energy to the plasma species to approach the substrate surface. However, warp *W_c_* starts to decrease when discharge power is higher than 160 W regardless of the oxygen flow rate.

**Figure 5:**
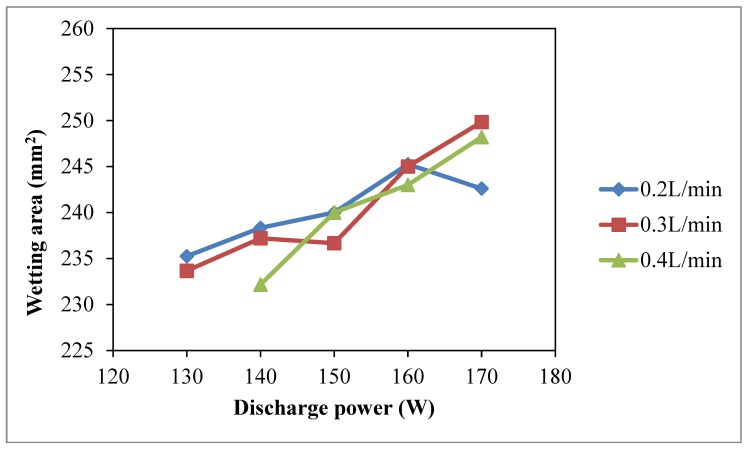
Effect of discharge power on wetting area (Discharge power: 130 W, 140 W, 150 W, 160 W and 170 W; Oxygen flow rate: 0.2 L/min, 0.3 L/min and 0.4 L/min; Jet travelling speed: 5 mm/s and Jet-to-substrate distance: 3 mm).

Wetting areas of plasma treated samples are larger than those of untreated samples (229.9 mm^2^) and the increasing trend of wetting area is shown in Figure 5, i.e., higher discharge power results in a larger wetting area. Figure 4 and Figure 5 show that discharge power of 170 W with oxygen flow rate of 0.2 L/min has a negative effect on water absorbency. In such high discharge power and low oxygen concentration condition, plasma species generated are less active [25] since oxygen molecules may gain more energy and become more active and then they may collide with each other easily before interacting with the material surface. Efficiency of reaction between active plasma species and the material surface is lower in such cases and as a result, there is a negative effect on water absorbency.

### 3.2. Oxygen Flow Rate

The effect of different oxygen flow rates (0.2 L/min, 0.3 L/min, 0.4 L/min, 0.5 L/min and 0.6 L/min) and discharge powers (150 W, 160 W and 170 W) on wettability of cotton fabric was investigated while the jet travelling speed (5 mm/s) and jet-to-substrate distance (3 mm) were kept constant. According to the instructions of the manufacturer, oxygen flow rate of 0.6 L/min is not recommended to be used when the discharge power is lower than 170 W because it generates unstable plasma effect. Therefore, no data of 0.6 L/min with discharge power of 150 W and 160 W are reported. 

**Figure 6:**
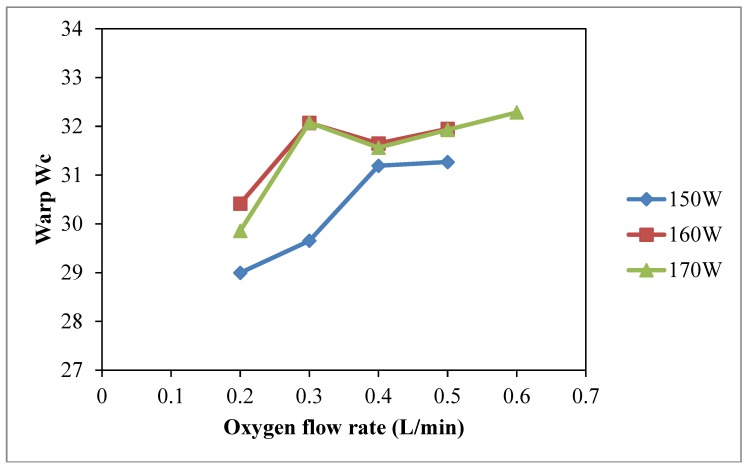
Effect of oxygen flow rate on warp *W_c_* (Oxygen flow rate: 0.2 L/min, 0.3 L/min, 0.4 L/min, 0.5 L/min and 0.6 L/min; Discharge power: 150 W, 160 W and 170 W; Jet travelling speed: 5 mm/s and Jet-to-substrate distance: 3 mm).

**Figure 7:**
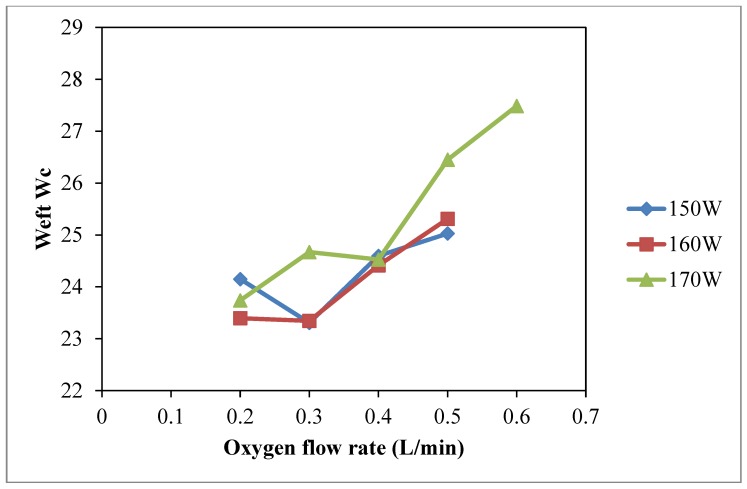
Effect of oxygen flow rate on weft *W_c_* (Oxygen flow rate: 0.2 L/min, 0.3 L/min, 0.4 L/min, 0.5 L/min and 0.6 L/min; Discharge power: 150 W, 160 W and 170 W; Jet travelling speed: 5 mm/s and Jet-to-substrate distance: 3 mm).

**Figure 8:**
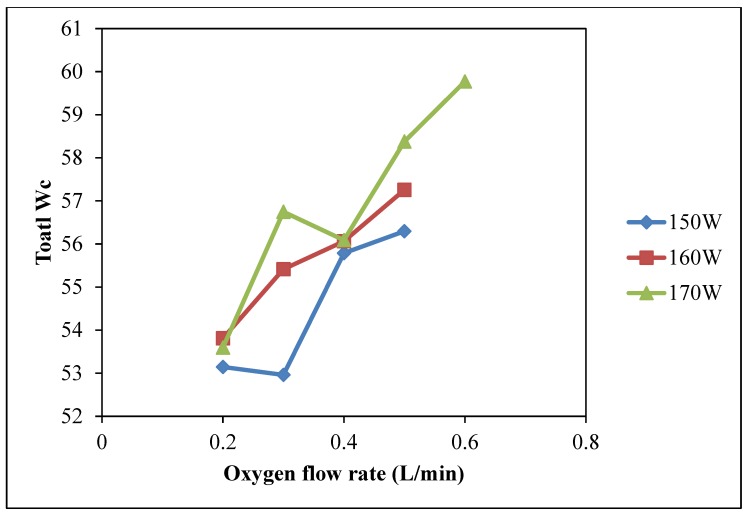
Effect of oxygen flow rate on total *W_c_* (Oxygen flow rate: 0.2 L/min, 0.3 L/min, 0.4 L/min, 0.5 L/min and 0.6 L/min; Discharge power: 150 W, 160 W and 170 W; Jet travelling speed: 5 mm/s and Jet-to-substrate distance: 3 mm).

**Figure 9:**
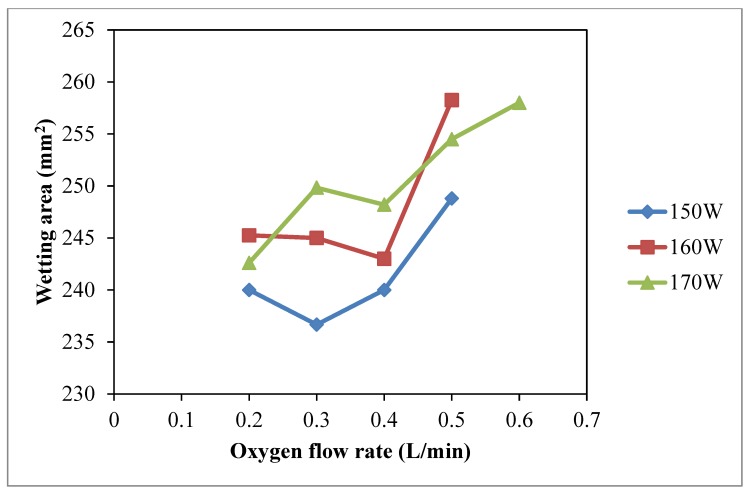
Effect of oxygen flow rate on wetting area (Oxygen flow rate: 0.2 L/min, 0.3 L/min, 0.4 L/min, 0.5 L/min and 0.6 L/min; Discharge power: 150 W, 160 W and 170 W; Jet travelling speed: 5 mm/s and Jet-to-substrate distance: 3 mm).

The results of warp *W_c_*, weft *W_c_*, total *W_c_* with different oxygen flow rates are shown in Figure 6, Figure 7 and Figure 8, respectively, and the effect of oxygen flow rate on the wetting area is shown in Figure 9. Figure 6 clearly shows that discharge power at 160 W and 170 W shows a better improvement on warp wicking behavior than discharge power of 150 W under the same flow rate of oxygen. However, the effect of discharge power of 160 W and 170 W on the warp wicking is not significantly different. Warp wicking increases when oxygen flow rate increases from 0.2 L/min to 0.3 L/min but the trend is flattened from 0.4 L/min to 0.6 L/min. This indicates that further change of discharge power from 160 W to 170 W does not further enhance the warp *W_c_* under the same oxygen flow rate. 

In case of weft wicking (Figure 7), it is suggested that active plasma species can be generated under high discharge power and oxygen flow rate which may have a better diffusion ability. The improved diffusion ability can cause a significant improvement of wicking performance in weft direction [10]. Since weft yarns are covered by the warp yarns, the diffusion ability of the energetic active plasma species may not be sufficient to interact with the weft yarn. Therefore, the warp *W_c_* is higher than weft *W_c_* under the same plasma treatment conditions. 

The increasing trends of water absorbency are shown in Figure 8 (Total *W_c_*) and Figure 9 (Wetting area) when oxygen flow rate is generally increasing under three different discharge power levels (150 W, 160 W and 170 W). Discharge power of 170 W with oxygen flow rate of 0.6 L/min and discharge power of 160 W with oxygen flow rate of 0.5 L/min yield the highest values of water wicking ability and wetting area [26]. Under high flow rate of oxygen, the oxygen population is high and more active plasma species are generated. Due to the effective collisions with sufficient energy with the fiber surface [27,28], better water absorption performance would be achieved by more etching and oxidization [22,23,24]. 

### 3.3. Jet Travelling Speed

The effect of plasma modification is highly correlated to concentration of active plasma species on the substrate surface [10]. The interaction of active plasma species on the textile fabric surface is based on the duration and travel distance of active plasma species that accumulate on surface [10,29]. Thus, the jet travelling speed is used to control the duration of the active plasma species accumulating on the cotton fabric surface in this study. To investigate the effect of jet travelling speed on wettability of cotton fabric, different jet travelling speeds (1 mm/s, 3 mm/s, 5 mm/s, 7 mm/s and 9 mm/s) and jet-to-substrate distances (3 mm, 5 mm and 7 mm) were used. The discharge power and oxygen flow rate were held at 150 W (to avoid thermal oxidation effect at high discharge power and unstable discharging [28]) and 0.4 L/min, respectively. 

**Figure 10:**
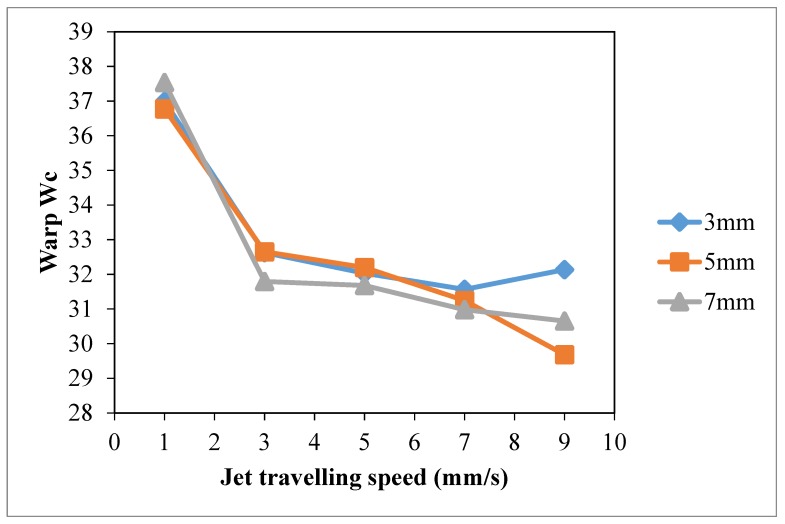
Effect of jet travelling speed on warp *W_c_* (Jet travelling speed: 1 mm/s, 3 mm/s, 5 mm/s, 7 mm/s and 9 mm/s; Jet-to-substrate distances: 3 mm, 5 mm and 7 mm; Discharge power: 150 W and Oxygen flow rate: 0.4 L/min).

**Figure 11:**
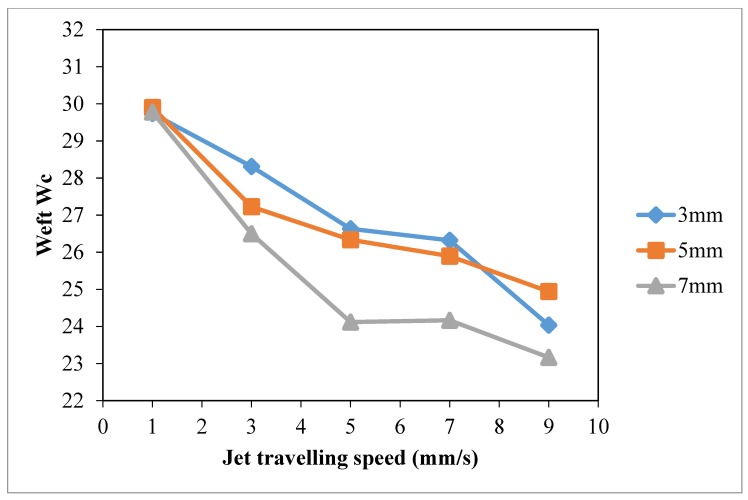
Effect of jet travelling speed on weft *W_c_* (Jet travelling speed: 1 mm/s, 3 mm/s, 5 mm/s, 7 mm/s and 9 mm/s; Jet-to-substrate distances: 3 mm, 5 mm and 7 mm; Discharge power: 150 W and Oxygen flow rate: 0.4 L/min).

**Figure 12:**
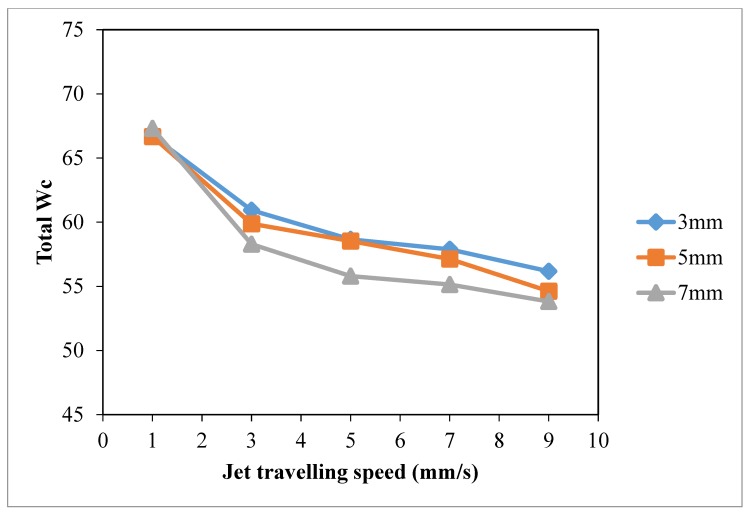
Effect of jet travelling speed on total *W_c_* (adapted from Ref. [29], with permission) (Jet travelling speed: 1 mm/s, 3 mm/s, 5 mm/s, 7 mm/s and 9 mm/s; Jet-to-substrate distances: 3 mm, 5 mm and 7 mm; Discharge power: 150 W and Oxygen flow rate: 0.4 L/min).

**Figure 13:**
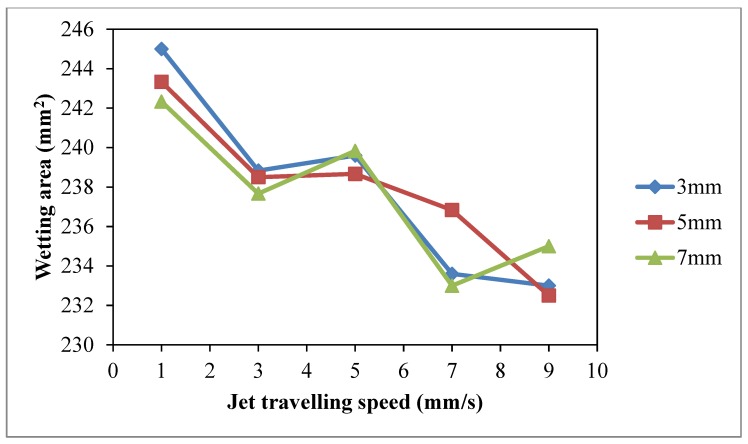
Effect of jet travelling speed on wetting area (Jet travelling speed: 1 mm/s, 3 mm/s, 5 mm/s, 7 mm/s and 9 mm/s; Jet-to-substrate distances: 3 mm, 5 mm and 7 mm; Discharge power: 150 W and Oxygen flow rate: 0.4 L/min).

Based on the results of warp wicking (Warp *W_c_*, Figure 10), weft wicking (Weft *W_c_*, Figure 11), total wicking (Total *W_c_*, Figure 12) and wetting area (Figure 13), slow jet travelling speed is preferable since this can provide better plasma modification results on cotton wettability. However, the same combinations of plasma operational parameters can have different effects on warp and weft wicking results. In warp direction, no significant improvement on wicking performance is observed under different combinations of jet-to-substrate distances (3 mm, 5 mm and 7 mm) and jet travelling speed (1 mm/s, 3 mm/s, 5 mm/s, 7 mm/s and 9 mm/s). On the other hand, weft wicking ability is influenced significantly by the combination of jet travelling speed and jet-to-substrate distance, as shown in Figure 11.

A higher total wicking (Total *W_c_*, Figure 12) and larger wetting area (Figure 13) are obtained with jet travelling speed of 1 mm/s regardless of the jet-to-substrate distance. Jet travelling speed of 1 mm/s is sufficient for accumulating adequate amount of active plasma species. However, increasing the jet travelling speed together with the jet-to-substrate distance has a negative effect on wicking behavior of plasma treated cotton (Figure 12). A similar effect has also been observed in the results of wetting area (Figure 13). With the increase of jet travelling speed and jet-to-substrate distance, the amount of active plasma species that interacts with substrate surface declines and hence the reduced wicking and wettability effect [29].

### 3.4. Jet-to-Substrate Distance

The effect of jet-to-substrate distance on wettability of cotton fabric was studied under constant discharge power of 150 W (to avoid thermal oxidation effect at high discharge power and unstable discharging [28]) and oxygen flow rate of 0.4 L/min. Different jet travelling speeds (1 mm/s, 5 mm/s, 9 mm/s) and jet-to-substrate distances (3 mm, 4 mm, 5 mm, 7 mm and 9 mm) were used. 

**Figure 14:**
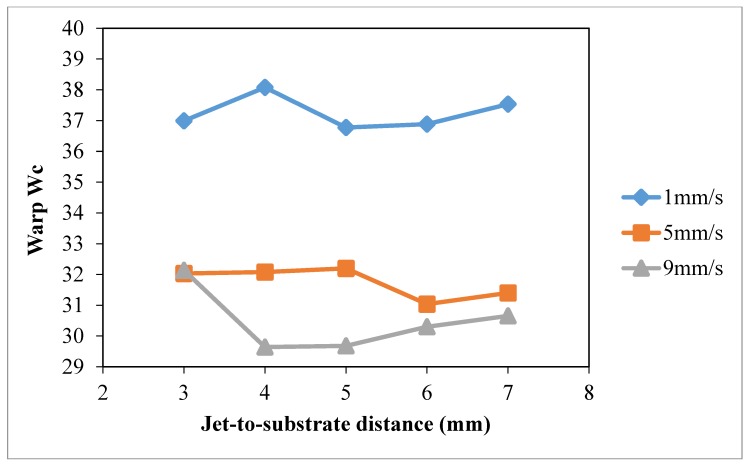
Effect of jet-to-substrate distance on warp *W_c_* (Jet-to-substrate distance: 3 mm, 4 mm, 5 mm, 7 mm and 9 mm; Jet travelling speed: 1 mm/s, 5 mm/s, 9 mm/s; Discharge power: 150 W and Oxygen flow rate: 0.4 L/min).

**Figure 15:**
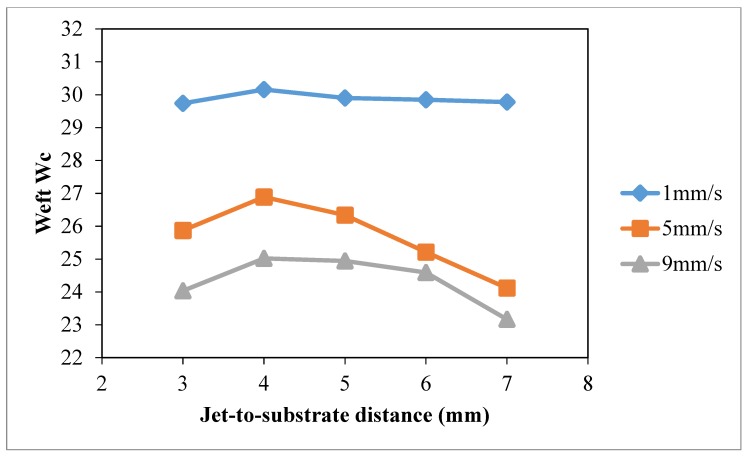
Effect of jet-to-substrate distance weft *W_c_* (Jet-to-substrate distance: 3 mm, 4 mm, 5 mm, 7 mm and 9 mm; Jet travelling speed: 1 mm/s, 5 mm/s, 9 mm/s; Discharge power: 150 W and Oxygen flow rate: 0.4 L/min).

**Figure 16:**
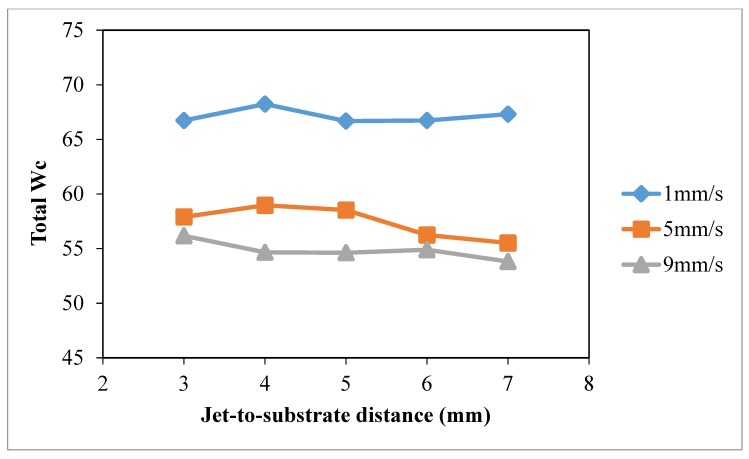
Effect of jet-to-substrate distance total *W_c_* (Jet-to-substrate distance: 3 mm, 4 mm, 5 mm, 7 mm and 9 mm; Jet travelling speed: 1 mm/s, 5 mm/s, 9 mm/s; Discharge power: 150 W and Oxygen flow rate: 0.4 L/min).

**Figure 17:**
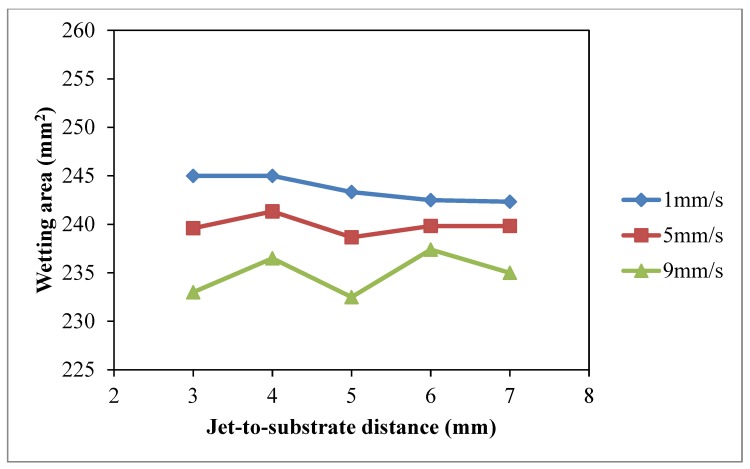
Effect of jet-to-substrate distance wetting area *W_c_* (Jet-to-substrate distance: 3 mm, 4 mm, 5 mm, 7 mm and 9 mm; Jet travelling speed: 1 mm/s, 5 mm/s, 9 mm/s; Discharge power: 150 W and Oxygen flow rate: 0.4 L/min).

The results of warp *W_c_*, weft *W_c_*, total *W_c_* and wetting area test with different jet-to-substrate distances are summarized in Figure 14, Figure 15, Figure 16 and Figure 17, respectively. Jet-to-substrate distance is defined as the perpendicular distance between plasma jet and the substrate located directly below it, which is also the distance active plasma species have to travel to reach the substrate surface. The travel distance of plasma active species can affect the efficiency of the atmospheric pressure plasma modification in terms of surface etching and polar functional groups formation [10,28]. 

The influence of jet-to-substrate distance on warp (Figure 14) and weft (Figure 15) direction cotton fibers depends on the relative jet travelling speed. With the use of jet travelling speed of 5 mm/s and 9 mm/s, variations of weft *W_c_* correlate more to jet-to-substrate distance. When comparing the three jet travelling speeds, a more significant improvement of total *W_c_* is obtained, as shown in Figure 16, with jet-to-substrate distance of 4 mm when the jet travelling speed is 1 mm/s or 5 mm/s. A small jet-to-substrate distance allows effective energy transfer from plasma active species to cotton surface with fewer collisions with air molecules or formation of ozone with oxygen [28,29,30,31,32]. In addition, a short travel distance (jet-to-substrate distance) favors active oxygen plasma species accumulation on cotton fabric when the treatment time is extremely short. As observed in drop test (Figure 17) wetting area is higher when jet-to-substrate distance is 4 mm in the case of jet travelling speed of 1 mm/s and 5 mm/s but the wetting area varies in the case of 9 mm/s. 

## 4. Conclusions

In this study, ready-to-dye cotton fabric was subjected to atmospheric pressure plasma treatment under different combinations of operations parameters: (1) discharge power; (2) flow rate of oxygen; (3) jet travelling speed; and (4) jet-to-substrate distance. Generally speaking, the atmospheric pressure plasma treatment can further enhance cotton fabric wettability in terms of wickability and wetting area. Experimental results reveal that the operational parameters interact with each other in terms of their effects on wettability of the cotton fabric. Therefore, this study provides significant technical data and information for developing atmospheric pressure plasma treatment in textile production.

## Figures and Tables

**Table 1:** Comparison of plasma treated and conventional chemical process (adapted from Ref. [5], with permission).

Parameter	Plasma Treated	Conventional Chemical Process
Solvent	None (gas phase)	Water
Energy	Electricity	Heat
Type of reaction	Complex	Simple
Deepness of the treatment	Very thin layer	Bulk of the fiber
New treatment equipment	Totally new	Slow evolution
Water and energy consumption	Low	High
Pollution	Very low	High

## References

[1] Morent R., De Geyter N., Verschuren J., De Clerck K., Kiekens P., Leys C. (2008). Non-thermal plasma treatment of textiles. Surf. Coat. Technol..

[2] Luo S., Van Ooik W.J. (2002). Surface modification of textile fibres for improvement of adhesion to polymeric matrices: A review. J. Adhes. Sci. Technol..

[3] Yasuda H. (1981). Glow discharge discharges polymerization. Macromol. Rev..

[4] Rakowski W. (1997). Plasma treatment of wool today, part I—Fibre properties, spinning and shrinkproofing. J. Soc. Dyers Colour..

[5] Buyle G., Heyse P., Ferreira I., Rausher H., Perucca M., Buyle G. (2010). Tuning the surface properties of textile materials. Plasma Technology for Hyperfunctional Surface.

[6] Karahan H.A., Özdoğan E. (2008). Improvements of surface functionality of cotton fibres by atmospheric plasma treatment. Fibers Polym..

[7] Leroux F., Campagne C., Perwuelz A., Gengembre L. (2008). Fluorocarbon nano-coating of polyester fabrics by atmospheric air plasma with aerosol. Surf. Coat. Technol..

[8] Leroux F., Perwuelz A., Campagne C., Behary N. (2006). Atmospheric air-plasma treatments of polyester textile structures. J. Adhes. Sci. Technol..

[9] Samanta K.K., Jassal M., Agrawal A.K. (2009). Improvement in water and oil absorbency of textile substrate by atmospheric pressure cold plasma treatment. Surf. Coat. Technol..

[10] Wang C.X., Qiu Y.P. (2007). Tow sided modification of wool fabric by atmospheric pressure plasma jet: Influence of processing parameter on plasma penetration. Surf. Coat. Technol..

[11] Hodak S.K., Supasai T., Paosawatyanyong B., Kamlangkla K., Pavarajarn V. (2008). Enhancement of the hydrophobicity of silk fabrics by SF_6_ plasma. Appl. Surf. Sci..

[12] Lei J., Shi M., Zhang J. (2000). Surface graft copolymerization of hydrogen silicon fluid onto fabric through corona discharge and water repellency of grafted fabric. Eur. Polym. J..

[13] Kan C.W., Lam C.F., Chan C.K., Ng S.P. (2014). Using atmospheric pressure plasma treatment for treating grey cotton fabric. Carbohydr. Polym..

[14] Cai Z.S., Qiu Y.P. (2006). The mechanism of air/oxygen/helium atmospheric plasma action on PVA. J. Appl. Polym. Sci..

[15] Jocic D., Vilchez S., Topalovic T., Molina R., Navarro A., Jovancic P., Julia M.R., Erra P. (2005). Effect of low-temperature plasma and chitosan treatment on wool dyeing with acid red 27. J. Appl. Polym. Sci..

[16] Ren C.S., Wang D.Z., Wang Y.N. (2008). Improvement of the graft and dyeability of linen by DBD treatment in ambient air. J. Mater. Process. Technol..

[17] Tokino S., Wakida T., Uchiyama H., Lee M. (1993). Laundering shrinkage of wool fabric treated with low-temperature plasmas under atmospheric pressure. J. Soc. Dyers Colour..

[18] Šimor M., Ráhel J., Černák M., Imahori Y., Štefečka M., Kando M. (2003). Atmospheric-pressure plasma treatment of polyester nonwoven fabrics for electroless plating. Surf. Coat. Technol..

[19] Szabová R., Černáková L., Wolfová M., Černák M. (2009). Coating of TiO_2_ nanoparticles on the plasma activated polypropylene fibres. Acta Chim. Slovaca.

[20] Zhou C.E., Kan C.W., Matinlinna J.P., Tsoi J.K.H. (2017). Regenerable antibacterial cotton fabric by plasma treatment with dimethylhydantoin: Antibacterial activity against *S. aureus*. Coatings.

[21] Zhou C.E., Kan C.W., Yuen C.W.M. (2015). Orthogonal analysis for rechargeable antimicrobial finishing of plasma pretreated cotton. Cellulose.

[22] Kan C.W., Man W.S. (2017). Enhancing dark shade pigment dyeing of cotton fabric with plasma treatment. Coatings.

[23] Kan C.W., Man W.S., Ng S.P. (2014). A study of pigment application on atmospheric pressure plasma treated cotton fabric. Fibers Polym..

[24] Kan C.W., Lo C.K.Y., Man W.S. (2016). Mini review—Environmentally friendly aspects in coloration. Color. Technol..

[25] Diamy A.M., Legrand J.C., Rybkin V.V., Smimov S.A. (2005). Experimental study and modelling of formation and decay of active species in an oxygen discharge. Contrib. Plasma Phys..

[26] Kan C.W., Kwong C.H., Ng S.P. (2016). Atmospheric pressure plasma surface treatment of rayon flock synthetic leather with tetramethylsilane. Appl. Sci..

[27] Jeong J.Y., Park J., Henins I., Babayan S.E., Tu V.J., Selwyn G.S., Ding G., Hicks R.F. (2000). Reaction chemistry in the afterglow of an oxygen-helium, atmospheric-pressure plasma. J. Phys. Chem. A.

[28] Schütze A., Jeong J.Y., Babayan S.E., Park J., Selwyn G.S., Hicks R.F. (1998). The atmospheric-pressure plasma jet: A review and comparison to other plasma sources. IEEE Trans. Plasma Sci..

[29] Man W.S., Kan C.W., Ng S.P. (2014). The use of atmospheric pressure plasma treatment on enhancing the pigment application to cotton fabric. Vacuum.

[30] Sun S.Y., Sun J., Yao L., Qiu Y.P. (2011). Wettability and sizing property improvement of raw cotton yarns treated with He/O_2_ atmospheric pressure plasma jet. Appl. Surf. Sci..

[31] Kan C.W., Lam C.F. (2018). Atmospheric pressure plasma treatment for grey cotton knitted fabric. Polymers.

[32] Kan C.W., Cheung H.F., Kooh F.M. (2017). An investigation of colour fading of sulphur-dyed cotton fabric by plasma treatment. Fibers Polym..

